# Definition and Structure of Body-Relatedness from the Perspective of Patients with Severe Somatoform Disorder and Their Therapists

**DOI:** 10.1371/journal.pone.0042534

**Published:** 2012-08-14

**Authors:** Hanneke Kalisvaart, Saskia van Broeckhuysen, Martina Bühring, Marianne B. Kool, Sandra van Dulmen, Rinie Geenen

**Affiliations:** 1 Altrecht Psychosomatic Medicine, Zeist, The Netherlands; 2 Department of Clinical and Health Psychology, Utrecht University, Utrecht, The Netherlands; 3 NIVEL (Netherlands Institute for Health Services Research), Utrecht, The Netherlands; University of Potsdam, Germany

## Abstract

**Background:**

How a patient is connected with one's body is core to rehabilitation of somatoform disorder but a common model to describe body-relatedness is missing. The aim of our study was to investigate the components and hierarchical structure of body-relatedness as perceived by patients with severe somatoform disorder and their therapists.

**Methods:**

Interviews with patients and therapists yielded statements about components of body-relatedness. Patients and therapists individually sorted these statements according to similarity. Hierarchical cluster analysis was applied to these sortings. Analysis of variance was used to compare the perceived importance of the statements between patients and therapists.

**Results:**

The hierarchical structure included 71 characteristics of body-relatedness. It consisted of three levels with eight clusters at the lowest level: 1) understanding, 2) acceptance, 3) adjustment, 4) respect for the body, 5) regulation, 6) confidence, 7) self-esteem, and 8) autonomy. The cluster ‘understanding’ was considered most important by patients and therapists. Patients valued ‘regulating the body’ more than therapists.

**Conclusion:**

According to patients with somatoform disorders and their therapists, body-relatedness includes awareness of the body and self by understanding, accepting and adjusting to bodily signals, by respecting and regulating the body, by confiding and esteeming oneself and by being autonomous. This definition and structure of body-relatedness may help professionals to improve interdisciplinary communication, assessment, and treatment, and it may help patients to better understand their symptoms and treatment. (German language abstract, Abstract S1; Spanish language abstract, Abstract S2).

## Introduction

Somatoform disorder is characterized by physical symptoms that suggest a general medical condition but are not fully explained by this condition or by the direct effects of a substance or another mental disorder [1]. When the whole spectrum from minor to severe somatoform disorder is considered, the prevalence in general practice is about 16 to 21 percent [2]. A distorted relation with one's body is core to somatoform disorder and treatment usually aims at changing this relation on a cognitive, emotional, and behavioral level. Terms such as body attitude, body schema, body experience, and body awareness have been used heterogeneously in literature as umbrella terms to refer to various aspects of body experience that are considered important in somatoform disorder [3], [4], [5], [6]. The terms that cover such various aspects as perception, cognition, emotion, awareness, and sometimes behavior, are mostly used to describe the current state of affairs, while patients and therapists are typically also concerned with the desired state of affairs. We use the term “body-relatedness” to refer to what a patient can learn in relation to the body when being in therapy.

Several theories emphasize the importance of the patients' cognitive, emotional, and behavioral relatedness to the body as a therapeutic target in somatoform disorder. Acceptance and commitment therapy focuses on the acceptance of the bodily symptoms [7], [8], cognitive behavioral therapy aims at optimal adaptation to bodily symptoms [9], [10], and still other therapies emphasize the importance of revealing the knowledge embedded in the body [11] and adaptive body awareness by non-judgmental mindfulness instead of hypervigilance [3]. These approaches may overlap with each other in their points of view and, together, cover a broad spectrum of ways one can relate to the body. However, an integrative model comprising these various components is missing [12].

Various components of body-relatedness have been emphasized in clinical literature. Treatment programs for somatoform disorders for instance commonly emphasize body-relatedness components like awareness, acceptance, expression of the self, pain management, and adaptation to impairment [13], [14], [15], [16], [17], [18]. Perceived body sensations, attention quality, attitude, and mind-body integration are seen as being of key importance for an appropriate questionnaire [3]. And in focus groups of expert practitioners and patients a shift in awareness of the body and negative emotions towards self regulation, self care and integration of mind, body and life context have been considered important [19]. Integrating these components in one definition could clarify the interrelationships between components of body-relatedness and their relative importance in somatoform disorder.

The aim of this study was to identify all relevant components comprising body-relatedness and the hierarchical structure and importance of these constituting components as perceived by patients with severe somatoform disorder and their therapists. Using the practice-based knowledge and points of view of both groups can result in a definition of body-relatedness that is used broadly in communication between disciplines and between patients and health care providers [5], [20], and it can give a common framework in assessment, goal setting, treatment, and research.

## Population and Methods

### Ethics Statement

The study was approved by the insititutional review board (CWO) of Altrecht Psychosomatic Medicine, Zeist, The Netherlands. All patients provided written informed consent.

### Participants

This study was conducted at Altrecht Psychosomatic Medicine, Zeist, The Netherlands, a specialized tertiary treatment center in which only the most severely impaired patients are examined and treated. The diagnosis according to DSM IV criteria was established by a multidisciplinary team of professionals. The main inclusion criterion in the current study was a severe somatoform disorder as the primary diagnosis according to DSM IV criteria with exception of hypochondria and body dysmorphic disorder. Hypochondrias and body dysmorphic disorder are not treated in our center, because it is debatable whether these are genuine somatoform disorders or could better be classified as obsessive-compulsive and related disorders [21]. Also excluded were patients with addiction, bipolar disorder, and psychosis as well as patients in a crisis situation requiring immediate attention and patients who were still under investigation with a specialized physician aside from Altrecht Psychosomatic Medicine. Comorbidity of other diagnoses [22] was allowed, as long as it was not considered to impede treatment of the somatoform disorder.

The data collection consisted of interviews (in 2004 and 2005) and a card sorting task (in 2010 and 2011). Hierarchical cluster analysis was applied to examine the characteristics and hierarchical structure of body-relatedness from the perspective of patients and their therapists.

A convenience sample of ten patients from our treatment center was selected by their therapists according to their availability and interview capability and invited to participate in the interviews: five patients (all female) who just started treatment and five patients (one male) who successfully ended their treatment (mean age 40 years, *SD* = 11, range 29–59). They all had at least secondary education. Eleven professionals (1 male, 10 female) with different disciplines participated (1 psychiatrist, 1 medical doctor, 2 physical therapists, 2 psychotherapists, 1 creative arts therapist, 1 body psychotherapist, 2 nurses, and 1 social worker). Their mean time of psychosomatic specialisation was 10 years (*SD* = 9, range 1.5–30).

For the card sorting task, other participants were invited than the ones who were interviewed. A call by letter among patients from clinic, day-clinic, and a psychotherapy group resulted in 21 patient volunteers (5 male, 14 female, 2 gender not noted). A call among professionals by e-mail or personally, resulted in twenty participants (3 male, 17 female). The mean age of the patients was 42 years (*SD* = 11, range 25–59), the mean time since the first symptoms was 12 years (*SD* = 11, range 1.5–40), and the mean time of specialized psychosomatic treatment was 1.3 years (*SD* = 1.7, range 0.15–7). They all had secondary or higher education. The professionals had a mean time of psychosomatic specialisation of 8 years (*SD* = 9, range 0.25–30).

### Interviews

Eight patients and all professionals were interviewed at Altrecht Psychosomatic Medicine and two patients were interviewed at their homes. The interviews were semi-structured and the duration was 30 to 60 minutes. The main question was: “What do you think are the most important issues a patient has to learn in relation to his/her body?” The participants were asked to explain their answers and to illustrate the meaning with concrete statements.

The interviews were summarized and returned to the participants who could correct the text. From the interviews all relevant statements regarding body-relatedness were extracted for the card sorting task. Statements that evidently could not be generalized to all people with psychosomatic disorder were removed (e.g., “discover why I had to suppress my body”) and overlapping statements were combined (e.g., “feel bodily signals” and “feel the body”). These statements were adjusted with respect to language and grammar and modified to statements fitting the phrase “A patient may learn…” (see Table S1). The selected statements were written down on separate cards and numbered.

### Card Sorting Task

The number of 20 participants is considered appropriate to obtain a variety of sortings [23]. Eight patients performed the card sorting task at Altrecht Psychosomatic Medicine and 14 performed the task at their homes. The professionals performed the task at Altrecht Psychosomatic Medicine. The duration was 45 to 60 minutes.

Research participants performed two card sorting tasks. First, they individually sorted the cards with the statements according to similarity, into piles that they gave labels. The following rules applied: all statements had to be placed in a pile; each statement could be placed in one pile only; each pile could contain 2 to 25 statements; and 4 to 20 piles could be formed.

In a second task, the participants individually sorted the cards with the statements based on the extent to which they considered them important for body-relatedness, defined as: what a patient may learn in relation to his/her body. The separate statements were rated from 1 (least important) to 5 (most important). The following rules applied: exactly five piles had to be formed from least to most important, statements had to be distributed equally across the five piles, all statements had to be placed in a pile, and each statement could be placed in one pile only. The results were written down on a score form by the participants. Not all participants had time to perform the second card sorting task. Nineteen patients (4 male, 13 female, 2 missing) and 12 professionals (2 male, 10 female) participated.

### Data Analysis

#### Hierarchical cluster analysis

Cluster analysis is a statistical technique to classify objects of a similar kind into clusters [24]. These clusters are organized hierarchically and can be graphically presented in a dendogram. Hierarchical cluster analysis (Ward's method, squared Euclidean distances) in the statistical software program SPSS, version 16.0 (SPSS, Chicago, IL), was used to classify the statements that were individually sorted by the participants according to their similarity. Statements that were sorted in the same pile because of similarity by many participants were grouped on the lowest level.The lower-order clusters that were the most closely related were grouped in higher-order clusters. These higher-order clusters were grouped in still higher-order clusters until there was a single highest-order cluster. The main criterion was that the separate lower-order clusters of statements should reflect distinct components of body-relatedness. To set the final number of lowest-order clusters, we used in the first stage top-down interpretation starting with two clusters, then three and so on until additional clusters did not yield new content. In the second stage, the contents of both a lower and a higher number of clusters were compared to finally decide on the number of clusters. The final hierarchical organization of the total group, with labels given to the clusters by consensus of three researchers (HK, SvB, RG), is graphically presented in a dendogram. Separate cluster solutions of the patients and professionals were compared to judge if these fitted the cluster solution of the total group.

#### Analysis of variance

For each statement a mean importance score across respondents was calculated. Moreover, the importance of clusters was derived by calculating mean importance scores of all the statements in a given cluster across respondents. These scores reflect the mean importance of the unweighted importance scores of statements in a cluster as perceived by the respondents. The differences in importance between the clusters and between the two groups (patients versus professionals) were analyzed with repeated measures analysis of variance.

## Results

### Interviews

The interviews yielded 68 statements about characteristics of body-relatedness from the patients and 49 from the professionals. Removing statements that could not be generalized and overlapping statements resulted in a final selection of 71 statements.

### Card Sorting Task

In the first sorting task, participants sorted the cards with statements according to similarity. There were large differences between participants in the number of piles they used to categorize the 71 statements. The number of piles across the participants varied from 4 to 14. Individual participants used 39 distinctive labels to describe the piles. Labels that were frequently chosen included terms like knowledge about the body, limitations and adjustment, acceptance, control and management, body and self awareness, and self-other. These multiple labels were used by the investigators to interpret the hierarchical cluster solution and to choose final labels for the clusters.

### Hierarchical Cluster Analysis

**Figure 1:**
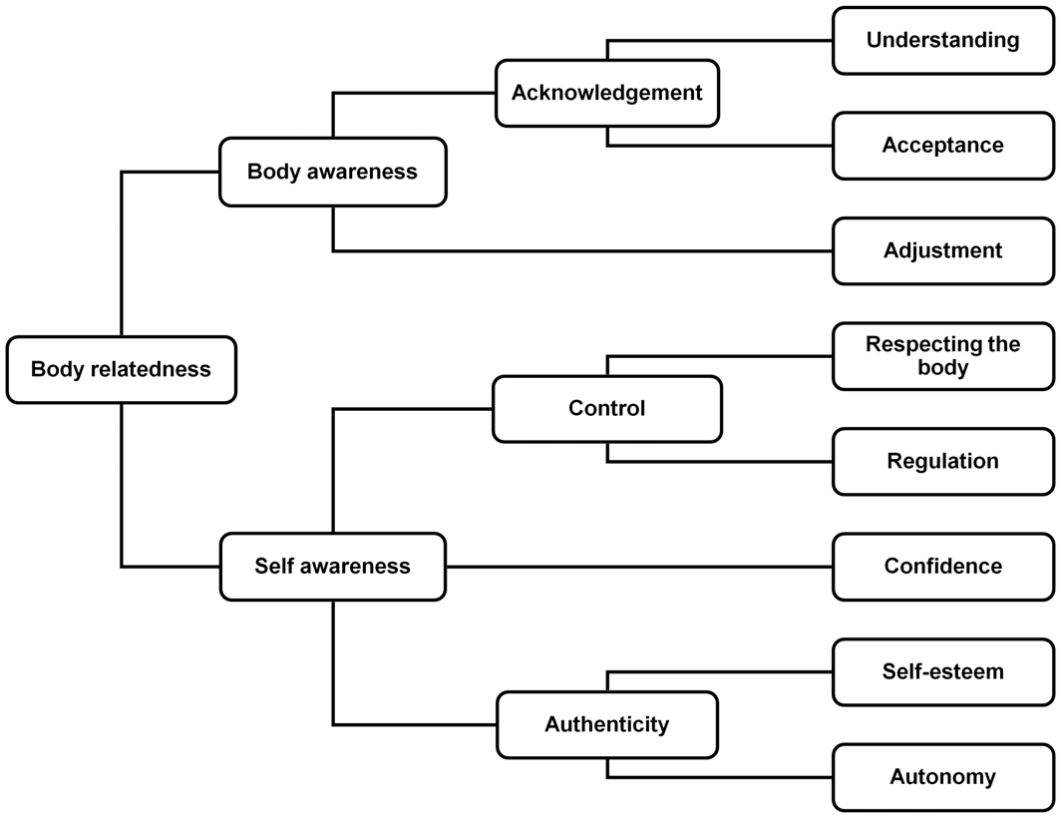
Figural representation of the hierarchical structure of components of body-relatedness according to patients and their therapists.

The outcome of the hierarchical cluster analysis structuring the 71 statements of the total group is shown in Figure 1. The statements included in the clusters are shown in Table S1. The structure consisted of three levels with eight components at the lowest level, three at the second, and two at the first. At the highest level the components were divided into ‘body awareness’ and ‘self-awareness’.

*Body awareness* consisted of 25 statements that mostly referred to the phenomological sense of body. Two clusters of statements at the lowest level referred to ‘acknowledgement’ of the body by ‘understanding’ and ‘accepting’ bodily signals. Examples of the 16 statements that covered understanding were: “…notice bodily signals” and “…get to know the body”. An example of the four statements about acceptance was: “…accept that one can do less than others”.

Next to these two acknowledgement clusters, the third cluster included in the broad body awareness domain of the hierarchical structure was ‘adjustment’. It comprised five statements like: “…adapt to what is possible” and “…work out what one is still capable of doing”.

*Self awareness* consisted of 56 statements that referred to strengthening the sense of self by ‘control over the body’, ‘confidence’ and ‘authenticity’. Control over the body consisted of four statements about ‘respecting the body’ and nine about ‘regulating’ one's body. An example of a respect statement was “…not see the body as a tool”. Regulation included for example “…be able to influence tiredness” and “…rediscover structure”.

The confidence cluster included statements like “…be satisfied” and “…trust the body”. Authenticity was divided into ‘self-esteem’ (8 statements) and ‘autonomy’ (19 statements). Self-esteem consisted of statements like “…express feelings” and “…feel respected”. Examples of the autonomy statements were “…discover what one likes” and “…dare to show one's limitations”.

### Group comparison

The structure of the sortings of the distinct groups was largely similar on the lower level: the clusters of patients and professionals included mostly similar statements with only some statements being placed in different clusters. On the highest level, however, the clusters of the professionals were split into awareness clusters (e.g. autonomy and acknowledgement) versus clusters related to further development (e.g. regulation and self-esteem) instead of the distinction between body- and self-awareness from the patients (and total group). Also the patients classified ‘control over the body’ as body awareness, contrary to the total group outcome where it was classified as self-awareness.

### Analysis of Variance

In the second sorting task, participants individually sorted the cards with regard to importance for body-relatedness. Table S1 shows the mean importance scores of the individual items. The five statements that were valued as most important (mean>3.80) were: “relax”, “listen to the body”, “notice bodily signals”, “experience a connection with oneself” and “be more aware of limits”. The statements that were considered least important (mean<2.15) were: “feel more masculine/feminine”, “reduce over-sensitivity”, “concentrate better”, “not let oneself be defined in terms of physical ailments”, and “be independent”.

Table 1 shows the mean scores of importance of the clusters for the patients and professionals, based on the hierarchical structure of both groups together. The scores of the total group varied from 2.59 (SD = .57) for regulation (which obtained a lower importance score (*p*<0.05, df = 14) than all other clusters except respect and self-esteem) to 3.44 (SD = .58) for understanding (which obtained a higher score than all self-awareness clusters (*p*<0.05, df = 14), except confidence).

**Table 1:** Mean scores (standard deviation) of importance of the clusters.

		Statistics			95% Confidence	
					Interval for Mean	
Clusters	Group	n	Mean	SD	Lower	Upper
					Bound	Bound
Understanding	Patients	19	3.31	.64	3.00	3.61
	Professionals	11	3.69	.36	3.45	3.94
	Total	30	3.45	.58	3.23	3.66
Acceptance	Patients	18	3.31	.74	2.94	3.67
	Professionals	12	3.12	.64	2.72	3.53
	Total	30	3.23	.69	2.97	3.49
Adjustment	Patients	19	3.11	.64	2.79	3.42
	Professionals	12	3.08	.62	2.69	3.48
	Total	31	3.10	.63	2.87	3.33
Respect	Patients	19	2.91	.58	2.63	3.19
	Professionals	12	2.98	.75	2.50	3.46
	Total	31	2.94	.64	2.70	3.17
Regulation	Patients	14	2.87	.51	2.58	3.17
	Professionals	12	2.26	.45	1.98	2.54
	Total	26	2.59	.57	2.36	2.82
Confidence	Patients	19	3.04	.49	2.80	3.27
	Professionals	12	3.10	.50	2.78	3.42
	Total	31	3.06	.49	2.88	3.24
Self-esteem	Patients	19	2.72	.78	2.34	3.09
	Professionals	12	2.59	.71	2.14	3.05
	Total	31	2.67	.74	2.40	2.94
Autonomy	Patients	19	3.01	.43	2.80	3.22
	Professionals	12	2.96	.39	2.71	3.21
	Total	31	2.99	.41	2.84	3.14

Patients valued regulation as more important than professionals (*p*<0.05, df = 1). The lower importance value attached to understanding by patients than professionals was not significant (*p* = 0.08, df = 1).

## Discussion

This study examined the components of body-relatedness and its hierarchical structure from the perspective of patients with severe somatoform disorder and their therapists. The results yield the following definition of body-relatedness in somatoform disorder: awareness of the body and self by understanding, accepting and adjusting to bodily signals, by respecting and regulating the body, by confiding and esteeming oneself and by being autonomous.

Our definition of body-relatedness includes cognitions, emotions, and behavior associated with the body. Consistent with the core problem of somatoform disorders it does not refer to appearance, as in eating disorders [25] or body dysmorphic disorder [26]. The multiple components of body-relatedness as specified in this study were categorized in two broad higher-order clusters including more body-oriented and more identity-related themes.

With respect to the higher-order cluster body awareness, understanding bodily signals was perceived as the most important learning goal in therapy (by patients as well as therapists), that is, to learn to listen to one's body and to know and recognize its signals. For most patients, however, this is a hard task because of fear, non-acceptance, or alexithymia [4], [8]. The other body-awareness components were accepting and adjusting. Accepting implies acknowledgement of pain and activity limitations, which, in case of a chronic condition, may induce a serious grieving process about giving up important things in life [27]. Core to adjustment is that the patient adapts his standards and does what he or she is capable of doing. This involves pacing activities [10] and abandoning “overactive” or “underachieving” lifestyles [9], [27]. To summarize, the body awareness components understanding, accepting and adjusting are perceived as important learning goals by professionals as well as by patients and these processes may take a long time to change.

The second higher-order cluster self-awareness comprised control, confidence, and authenticity. As for the control cluster, the body-mind relation is evident in its main components respecting and regulating the body. Components of the respecting clusters such as not seeing the body as a tool and not letting oneself be defined in terms of physical ailments reflect that patients can learn to less objectify their body and unite body and mind towards an indivisible integrity [19]. The importance attached to regulation is a little bit lower than the importance attached to other components, but patients think it is more important than professionals do. Their need to have a sense of control over their tired, painful or otherwise uncomfortable body might be underestimated by professionals who have the experience that listening and adjusting to the body will lead to better regulation. Perhaps the topic of regulation should get more attention at the start of treatment in order to motivate patients not only to try to control their body but also to listen to its signals.

The other self-awareness components, confidence, self-esteem and autonomy, emphasize how strong the connection with one's body is related to identity and personality [28]. The development of emotional awareness starts with the experience of physical sensations and action tendencies, resulting in distinction of emotions and the capacity to appreciate complexity in the experiences of self and other [4]. The cluster confidence refers to positive bodily feelings and trust that most patients have lost due to the problems with their body. Self-esteem ameliorates when patients feel respected and dare to express their feelings, even if these feelings concern tiredness or loss. If patients learn and dare to distinguish themselves from others instead of trying to meet expectations, they may perhaps change to a higher level of emotional awareness, appreciating the complex experience of self and other [4]. These self-awareness components that are mentioned in the literature as expression of the self [15], attitude [3], self care [19], and emotional awareness [4], emphasize the importance of a positive feeling about the unique self to overcome the difficulties of somatoform disorder.

Comparing the structure of the sorting of patients and professionals, a difference appears on the higher level where patients make a body-self split while professionals distinguish overall awareness from development. One can wonder if the duality in the total model is acceptable for professionals who emphasize the unity of body and mind. The model reflects a Western way of thinking about the body that professionals may encounter in most of their patients and in colleagues who are not specialized in somatoform disorder. However, since the total model reflects body-self dualism as well as body-self unity, it provides a tool to communicate about body-relatedness and to emphasize that it is important to integrate body and self.

Although the different components of body-relatedness are interrelated, the definition can serve well to decide which components in the treatment of individual patients should be emphasized. It provides a model that can be used as a checklist in assessment, therapeutic goalsetting, or evaluation and it might offer input to construct a questionnaire. However, as self-report questionnaires only measure components that the client is aware of and is willing to tell, the validity of a questionnaire for body-relatedness will likely be low. An interview and nonverbal observations by specialized therapists will provide more information about body-relatedness than self-reports of patients. Although this study did not focus on a specific method to ameliorate body-relatedness, the definition suggests that an integrative, multimodal approach is preferable. The model can be applied from different theoretical and clinical viewpoints.

One strength of the current study is that both patients and professionals were seen as experts who from their own experiences and perspectives specified the components of body-relatedness. Another strength is the use of both qualitative and quantitative methods allowing a description beyond the subjective interpretation of researchers. A limitation of the current study may be that the wording of statements could have influenced the sortings. Also, there was no control on the score forms that were filled out at home, resulting in missing values (for example concerning gender). With respect to external validity, the results of this study do not generalize beyond predominantly female patients with severe somatoform disorder and their therapists or people from a Dutch (Western) culture. Comparable studies in general practice or hospitals and in other countries should reveal whether the components of body-relatedness can be generalized to other groups.

In conclusion, the present study identified the components and hierarchical structure of body-relatedness from the perspective of patients with severe somatoform disorder and their therapists. The findings give direction to assessment, therapeutic goal setting, evaluation, and development of questionnaires and observation instruments, and may ameliorate communication between disciplines, which can lead to improved therapeutic targets in this difficult-to-treat patient group.

## Supporting Information

Table S1 **Clusters and statements.**
(DOC)

Abstract S1 **German abstract.**
(DOC)

Abstract S2 **Spanish abstract.**
(DOC)
