# Supplementary material for: Definition and Structure of Body-Relatedness from the Perspective of Patients with Severe Somatoform Disorder and Their Therapists
Source: PLoS One. 2012 Aug 14;7(8):e42534. doi: 10.1371/journal.pone.0042534 (PMC3419208; doi:10.1371/journal.pone.0042534)
Supplement: Table S1 — Clusters and statements. (DOC) [file pone.0042534.s001.doc]

Table S1. Clusters and statements

|  |  | **Importance attached to the statements** | |
| --- | --- | --- | --- |
|  |  | **(range 1-5)** | |
| **Clusters** | **Statements** | **Mean patients** | **Mean professionals** |
|  | (preceded by the words “A patient may learn…”) | **(n=19)** | **(n=12)** |
| Understanding | to acknowledge his/her body. | 3.53 | 3.25 |
|  | that physical sensations are signs of psychological processes. | 3.26 | 3.83 |
|  | to discover the meaning of body language. | 2.84 | 2.92 |
|  | to know what his/her body is capable of. | 3.11 | 3.00 |
|  | to know that his/her body has limits. | 3.47 | 3.55 |
|  | to understand physical functioning. | 2.95 | 3.08 |
|  | that psychological stress can exacerbate symptoms. | 3.26 | 3.92 |
|  | to get to know his/her body. | 2.95 | 4.25 |
|  | to understand bodily signals. | 3.42 | 3.50 |
|  | to take a break on time. | 3.42 | 3.17 |
|  | to notice bodily signals. | 3.42 | 4.50 |
|  | to take bodily signals seriously. | 3.58 | 4.00 |
|  | to listen to his/her body. | 3.58 | 4.33 |
|  | to take responsibility for his/her own body. | 3.21 | 3.92 |
|  | to respond to bodily signals. | 3.26 | 3.83 |
|  | to be more aware of his/her limits. | 3.63 | 4.08 |
| Acceptance | to acknowledge his/her limitations. | 3.28 | 3.50 |
|  | to accept his/her body | 3.63 | 3.25 |
|  | to tolerate pain. | 2.74 | 2.75 |
|  | to accept that he/she can do less than others. | 3.53 | 3.00 |
| Adjustment | to adapt to what is possible. | 2.74 | 2.92 |
|  | to lower goals. | 3.11 | 3.42 |
|  | to indicate what he/she can no longer do. | 3.63 | 3.00 |
|  | to take on less. | 2.89 | 3.17 |
|  | to work out what he/she is still capable of doing. | 3.16 | 2.92 |
| Respect for | to accept physical symptoms. | 3.32 | 2.92 |
| his/her body | to not see the body as a tool. | 3.05 | 3.00 |
|  | to not let oneself be defined in terms of physical ailments. | 2.26 | 1.92 |
|  | to deal appropriately with bodily signals. | 3.00 | 4.08 |
| Regulation | to reduce over-sensitivity. | 2.12 | 1.67 |
|  | to concentrate better. | 2.22 | 1.50 |
|  | to rediscover structure. | 2.94 | 3.25 |
|  | to deal with daily life. | 2.74 | 2.75 |
|  | to be able to influence tiredness. | 2.84 | 1.83 |
|  | to regulate breathing. | 2.84 | 2.17 |
|  | to relax. | 4.11 | 3.67 |
|  | to be able to influence pain. | 2.84 | 1.83 |
|  | to increase physical fitness. | 3.06 | 1.67 |
| Confidence | body-mind connection. | 2.84 | 4.17 |
|  | to be satisfied. | 2.63 | 2.42 |
|  | to trust his/her own body. | 3.74 | 3.58 |
|  | to think about oneself. | 2.89 | 2.75 |
|  | to be less insecure and anxious. | 3.00 | 2.08 |
|  | to act on his/her feelings. | 3.11 | 3.58 |
| Self-esteem | to feel respected. | 2.47 | 2.00 |
|  | to express feelings. | 3.58 | 3.67 |
|  | to be independent. | 2.21 | 2.00 |
|  | to voice feelings. | 3.05 | 3.42 |
|  | to feel good about oneself. | 3.00 | 2.33 |
|  | to enjoy. | 2.74 | 2.75 |
|  | to be oneself. | 3.11 | 3.17 |
|  | to feel more masculine/feminine. | 1.58 | 1.42 |
| Autonomy | to ask for attention. | 3.32 | 3.00 |
|  | to discover what he/she likes. | 2.79 | 2.33 |
|  | to experience a connection with oneself. | 3.79 | 3.92 |
|  | to dwell on oneself. | 3.05 | 3.75 |
|  | to experience oneself as a whole. | 2.63 | 3.42 |
|  | to see oneself as separate from others. | 2.58 | 2.42 |
|  | to dare to trust his/her own feelings. | 3.42 | 3.67 |
|  | to accept who he/she is. | 3.32 | 3.08 |
|  | to be more open. | 2.63 | 2.64 |
|  | to dare to show his/her limitations. | 3.47 | 3.08 |
|  | to say that he/she can’t do something. | 2.47 | 3.33 |
|  | to relax in the company of others. | 2.95 | 2.33 |
|  | to accept attention from others. | 2.89 | 2.27 |
|  | to be less afraid of the reaction of others. | 2.58 | 2.08 |
|  | to take up space. | 3.67 | 2.75 |
|  | that he/she can make choices. | 2.74 | 3.08 |
|  | to find a balance between looking after oneself and looking |  |  |
|  | after others. | 3.21 | 3.17 |
|  | to be able to tell his/her own story. | 2.58 | 2.75 |
|  | to deal with the person he/she has become. | 3.16 | 3.08 |
