# Supplementary material for: Definition and Structure of Body-Relatedness from the Perspective of Patients with Severe Somatoform Disorder and Their Therapists
Source: PLoS One. 2012 Aug 14;7(8):e42534. doi: 10.1371/journal.pone.0042534 (PMC3419208; doi:10.1371/journal.pone.0042534)
Supplement: Abstract S1 — German abstract. (DOC) [file pone.0042534.s002.doc]

Struktur und Definition von 'body-relatedness' bei somatoformen Störungen aus der Perspektive von Patienten und ihren Therapeuten

Die Beziehung zum Körper ist wichtig in der Rehabilitation von (Menschen mit) somatoformen Störungen; ein brauchbares  Modell zur Beschreibung von 'body-relatedness' jedoch fehlt. Ziel unserer Studie war es, die Eigenschaften und hierarchischen Struktur von 'body-relatedness', zu untersuchen, wie sie  von Patienten mit ernsten somatoformen Störungen und von ihren Therapeuten erlebt werden.

Interviews mit Patienten und Therapeuten führten zu Beschreibungen, die  für 'body-relatedness' charakteristisch sind. Patienten und Therapeuten ordneten diese Beschreibungen zu  unabhängig  voneinander auf Übereinstimmungen. Eine hierarchische Clusteranalyse wurde bei dieser Sortierung ausgeführt. Durch eine Varianzanalyse wurde die Wichtigkeit der Beschreibungen  zwischen  Patienten und Therapeuten verglichen.

Die hierarchische Struktur ergab 71 Charakteristiken von 'body-relatedness', bestehend aus drei Ebenen mit acht Einheiten, wobei die folgenden Niveaus für die Praxis die meiste Bedeutung haben:: 1) Verstehen, 2) Akzeptanz, 3) Begrenzung, 4) den Körper respektieren, 5) den Körper regulieren, 6) Vertrauen, 7) Selbstachtung, 8) Autonomie. 'Verstehen' wurde als am wichtigsten erachtet. Patienten bewerteten 'den Körper regulieren' wichtiger als es Therapeuten taten.

'Body-relatedness' weckt  gemäß der Patienten mit somatoformen Störungen und ihren Therapeuten ein Bewusstsein des Körpers und des Selbst, wodurch sie lernen, ihre  Körpersignale zu akzeptieren und zuzuordnen, den Körper zu respektieren und zu regulieren und sich selbst hierin zu vertrauen, Wert zu schätzen und sich von anderen zu unterscheiden

Das Konzept  von 'body-relatedness' kann Fachleute dabei unterstützen, ihre interdisziplinäre Kommunikation, Diagnostik und Behandlung zu verbessern.  Patienten kann es helfen, ihre Symptomatik und deren Behandlung besser zu verstehen.
