# Supplementary material for: Definition and Structure of Body-Relatedness from the Perspective of Patients with Severe Somatoform Disorder and Their Therapists
Source: PLoS One. 2012 Aug 14;7(8):e42534. doi: 10.1371/journal.pone.0042534 (PMC3419208; doi:10.1371/journal.pone.0042534)
Supplement: Abstract S2 — Spanish abstract. (DOC) [file pone.0042534.s003.doc]

La manera en que un paciente esta conectado con su cuerpo es la base de la rehabilitación del trastorno somatoforme. Lo que falta es un modelo común para describir la relación con el cuerpo. El objetivo de nuestro estudio fue el investigar los componentes y la estructura jerárquica del cuerpo en relación a como era percibido por los pacientes con trastorno somatoforme severo y sus terapeutas.

**Métodos:** Entrevistas con pacientes y terapeutas produjeron declaraciones acerca de los componentes en relación con el cuerpo. Pacientes y los terapeutas individualmente sortearon estas declaraciones según su similaridad. Jerárquicamente el conjunto de análisis fue aplicado a estas clasificaciones. El análisis de variación fue utilizado para comparar la importancia de lo percibido de las declaraciones entre pacientes y terapeutas.

**Resultados:** La estructura jerárquica  incluye las 71 características en relación al cuerpo. Consiste en tres niveles con ocho grupos en el nivel mas bajo: 1. Entendimiento, 2. Aceptación, 3. Adaptación 4. Respeto por el cuerpo, 5. Regulación, 6. Confianza, 7. Autoestima y 8. Autonomía. El grupo "entendimiento" fue considerado el más importante por los pacientes y los terapeutas. Los pacientes valoraron "regular el cuerpo" más que los terapeutas.

**Conclusión:** En función de pacientes con trastornos somatomorfos y sus terapeutas, en relación al cuerpo incluye la toma de conciencia del cuerpo y el entendimiento hacia uno mismo, aceptando y ajustando a señales corporales, respetando y regulando el cuerpo, y confiando, estimando y distingüendo a uno mismo. Esta definición y estructura de la relación del paciente con su cuerpo podría ayudar a los profesionales para mejorar la comunicación interdisciplinar, sus conclusiones y tratamiento, y podría ayudar comprender a los pacientes mejor sus síntomas y tratamiento.
